# Associations Between Violence and Psychological Distress Among Women Experiencing Homelessness: A Cross-Sectional Study

**DOI:** 10.1177/10778012251351935

**Published:** 2025-06-24

**Authors:** Anna Klarare, Elisabet Mattsson, Sophie Nadia Gaber, Penny Rapaport, Andreas Rosenblad

**Affiliations:** 1 The Connell School of Nursing, Boston College, Chestnut Hill, Boston, MA, USA; 2 Department of Women's and Children's Health, Digital Health and Mental Health Unit, 8097 Uppsala University, Uppsala, Sweden; 3 Department of Healthcare Sciences, Marie Cederschiöld University, Stockholm, Sweden; 4 Division of Psychiatry, Faculty of Brain Sciences, University College London, London, UK; 5 Department of Statistics, 8097 Uppsala University, Uppsala, Sweden; 6 Department of Medical Sciences, Division of Clinical Diabetology and Metabolism, 8097 Uppsala University, Uppsala, Sweden; 7 Department of Neurobiology, Care Sciences and Society, Division of Family Medicine and Primary Care, Karolinska Institutet, Stockholm, Sweden

**Keywords:** homelessness, psychological distress, trauma-informed care, violence, women

## Abstract

This study examines the association between violence exposure and psychological distress among women experiencing homelessness (*n* = 41). Using a cross-sectional survey design, we assessed emotional, physical, and sexual violence perpetrated by both close (e.g., intimate partners, family members) and non-close (e.g., acquaintances, strangers) individuals and their impact on mental well-being. Findings indicate high levels of violence, with greater exposure linked to increased distress. Each form of violence in close relationships was independently associated with distress, highlighting its broad health consequences. Addressing the significant burden of violence among women experiencing homelessness requires both healthcare recognition and structural interventions. Comprehensive, trauma-informed approaches are essential to mitigate the impact of violence on psychological well-being.

## Introduction

Addressing violence against women experiencing homelessness requires a nuanced understanding of the intersection between their vulnerable circumstances and the broader public health issues highlighted by the World Health Organization (WHO). The WHO emphasizes the severity of violence against women, categorizing it as both a public health concern and a violation of fundamental human rights (WHO, 2013, 2021). According to the United Nations (UN) (1993) definition, violence against women encompasses any form of gender-based violence leading to physical, sexual, or psychological harm or suffering, including acts of violence, threats, coercion, or arbitrary restrictions of freedom, in both public and private spheres.

A distinction is often made between violence perpetrated within close versus non-close relationships (WHO, 2021). Close relationships refer to interpersonal ties characterized by emotional intimacy and trust, such as those with intimate partners or family members. In contrast, non-close relationships include acquaintances, strangers, or individuals with whom the victim does not share an intimate bond. This distinction is important because prior research shows that violence occurring within close relationships is often associated with more severe emotional and psychological consequences than violence perpetrated by strangers or acquaintances (Hullenaar et al., 2022). The emotional betrayal involved in close relationship violence may intensify trauma, complicate recovery, and increase long-term psychological burden (Briere & Jordan, 2004). Furthermore, structural and social vulnerabilities, such as poverty, social inequality, and lack of support, can exacerbate these effects, particularly in marginalized populations. Given that women experiencing homelessness often lack both formal and informal support systems, understanding the role of perpetrator relationship is essential for informing trauma-sensitive interventions.

Globally, the WHO estimates that approximately one in three women experience physical and/or sexual violence, either from an intimate partner, a non-partner, or both (WHO, 2021). Most of this violence occurs within intimate partner relationships, affecting about 27% of women aged 15–49 who have been in a relationship (WHO, 2021). In addition, 6% of women globally report having been sexually assaulted by a non-partner, although data on non-partner violence remain limited. Primarily perpetrated by men (Walby et al., 2016), these forms of violence have serious short- and long-term health consequences, adversely affecting physical, mental, sexual, and reproductive health (Dworkin et al., 2017; Pines, 2017). Women exposed to such violence may suffer physical injuries (de Souza Cantão et al., 2024) as well as adverse mental health outcomes, including depression, posttraumatic stress disorder (PTSD), and suicidality (Dworkin, 2020; White et al., 2024). Importantly, violence against women also has economic and societal repercussions, extending beyond personal suffering to pose substantial social and economic challenges for societies worldwide (WHO, 2021). Research also suggests that individuals who have experienced or witnessed violence in their childhood or adolescence may be more likely to engage in violent behavior as adults (Widom, 2017).

The intersection of violence and homelessness is critical, as women experiencing unstable housing are disproportionately affected by health inequalities (Aldridge et al., 2018; Tweed et al., 2021) and trimorbidity (Fazel et al., 2014; Vickery et al., 2021). Trimorbidity, which refers to the co-occurrence of physical and mental health issues along with substance use disorder, has been linked to premature death (Aldridge et al., 2019), while the burden of PTSD further exacerbates existing health problems (Beijer et al., 2018; Roze et al., 2020). This complex interplay of health concerns not only complicates the daily lives of women experiencing homelessness (Bretherton, 2020; Duke & Searby, 2019) but is also intensified by social determinants, as health outcomes tend to worsen with lower sociodemographic status (Marmot et al., 2012).

In underserved populations, including women experiencing homelessness, the impact of violence is particularly harsh given their complex health and social care needs (Beijer et al., 2018; Fazel et al., 2014; Riley et al., 2020; Tinland et al., 2018). Gender-related factors, such as poverty, family conflict, lack of childcare (Andermann et al., 2021; Phipps et al., 2019), and intimate partner violence (Giano et al., 2020), significantly contribute to homelessness among women. Recent studies show that 92% of women experiencing homelessness have been exposed to violence before losing their housing, and being female is a major predictor of experiencing violence while without stable housing (Calvo et al., 2022). Rates of violence against women experiencing homelessness are significantly higher than in the general population (Riley et al., 2020) and compared to housed women (Beijer et al., 2018). Contributing factors such as sexual exploitation, violent victimization, and intimate partner violence (Li & Urada, 2020) create a complex trauma history that exacerbates the cumulative impact of these experiences (Duke & Searby, 2019; Phipps et al., 2019). Despite contending with serious mental and physical health concerns, women experiencing homelessness face significant barriers to accessing necessary care. The literature highlights the challenges in obtaining treatment for mental and physical issues among women facing homelessness (Kneck et al., 2021; Phipps et al., 2019).

Existing research shows that women experiencing homelessness are particularly vulnerable to the psychological impacts of violence (Beijer et al., 2018; Bretherton, 2020; Riley et al., 2020; Tinland et al., 2018). They are more frequently exposed to repeated violence and PTSD compared to housed women (Beijer et al., 2018) and have higher rates of depression and suicidal ideation than men in similar situations (Tinland et al., 2018). However, prior studies have often treated violence as a singular phenomenon, failing to examine how different forms of violence—emotional, physical, and sexual—interact and contribute to psychological distress. This gap in understanding limits the development of trauma-informed, gender-sensitive mental health interventions tailored to women experiencing homelessness (Anderson et al., 2024).

Previous research has demonstrated that different forms of violence have varying impacts on women's mental well-being. For instance, physical and sexual violence are associated with heightened psychological distress, particularly when perpetrated within close relationships, while psychological violence and controlling behaviors can, in some cases, have more persistent effects than physical violence (Richardson et al., 2020). Additionally, cumulative exposure to multiple forms of violence exacerbates psychological trauma among women (Hisasue et al., 2020; Vázquez et al., 2025); however, this dynamic has not been sufficiently explored in the context of women experiencing homelessness (Anderson et al., 2024).

The present study aimed to address these research gaps by examining how different forms of violence—emotional, physical, and sexual—are associated with psychological distress among women experiencing homelessness. In doing so, we consider violence perpetrated by both close and non-close individuals, to gain a broader understanding of how different sources of violence may relate to psychological outcomes. To this end, we hypothesize that emotional, physical, and sexual violence, regardless of the perpetrator, are associated with psychological distress in women experiencing homelessness. Furthermore, we hypothesize that witnessing violence against a close person during childhood, as well as engaging in violent behavior as an adult, are both associated with psychological distress in this vulnerable population.

## Methods

This study employed a cross-sectional survey design and analyzed primary quantitative data collected through standardized questionnaires, as part of a larger project focused on promoting inclusion health among women experiencing homelessness. Inclusion health is a service, research, and policy agenda aimed at addressing significant health disparities and the reduced life expectancy experienced by marginalized groups through collaborative efforts across sectors—nationally and internationally (Aldridge et al., 2018).

### Setting

In 2023, more than 27,000 individuals (32% women) with either Swedish citizenship or temporary/permanent residence permits were reported to be experiencing homelessness in Sweden (Socialstyrelsen [The National Board of Health and Welfare], 2024). Homelessness is most prevalent in larger municipalities, particularly in urban areas with at least 70,000 residents. Participants were recruited from a primary healthcare center (PHC) in Stockholm County (population 2.4 million) that primarily serves people experiencing homelessness. The PHC is located in Stockholm, the capital of Sweden, and shares a building with social services and a citizens’ service office, where individuals can receive support from social workers and access computers and phones.

The PHC receives approximately 14,000 annual visits from 1,300 patients, with about 40% of these being women experiencing homelessness. It is a privately run but publicly funded facility, operating on weekdays from 8:00 to 17:00, and offers both pre-booked appointments and drop-in services. The PHC provides general healthcare, psychiatric care, and substance use disorder treatment, along with specialized services including dental care, podiatric care, and reproductive health services. It maintains a close collaboration with social services, facilitating coordinated care and support for individuals with complex health and social needs.

The PHC shares a waiting room with social services. It is a busy area, with people moving in and out throughout the day for various reasons beyond seeking services from the PHC or social services. Some visit to use the restroom, access the citizens’ service office during drop-in hours, or simply get water, without being formally registered as patients or clients.

### Participants and Data Collection

A convenience sample of women experiencing homelessness (*n* = 41) was recruited for this study. The sample size was calculated for the larger project, based on the overarching aim of being able to detect a 10-point difference in the Caring Behaviors Inventory-24 score between women experiencing homelessness, registered nurses, and nursing students, assuming a standard deviation (SD) of 15 points. Using the Mann–Whitney *U* test with a two-sided null hypothesis (α = .05 and power = 0.80), the study aimed to recruit at least 35 women experiencing homelessness (Gaber et al., 2022).

Inclusion criteria were Swedish-speaking women, aged 18 years or older, with experience of one or more categories of homelessness based on the *Typology of Homelessness and Housing Exclusion*, that is, rooflessness (e.g., without any kind of shelter, sleeping rough); houselessness (e.g., with a place to sleep but temporary in institutions or shelters); living in an insecure accommodation (e.g., at risk of severe exclusion for reasons such as insecure tenancies); or living in an inadequate accommodation (e.g., in unfit housing) (European Federation of National Organisations Working with the Homeless [FEANTSA], 2017). Exclusion criteria included women visibly displaying severe anxiety or distress, as well as those exhibiting violent or aggressive behavior. In cases of uncertainty, PHC staff could be consulted.

All data were collected by a single female research assistant with prior experience working with women experiencing homelessness. She had previously worked at the PHC as an assistant nurse and was familiar to many of the women. Data collection began in October and November 2019 but was halted due to the COVID-19 pandemic. It resumed between September and December 2020. During the recruitment period, the research assistant spent approximately 90 hours at the PHC.

Potential participants were approached in the waiting area by the research assistant, regardless of whether they were previously registered at the PHC or with social services. If a woman showed interest in participating in the study, both verbal and written information about the study were provided in an adjoining meeting room. The women were given time to ask questions and discuss any concerns related to the study. However, due to privacy concerns, we did not record the number of individuals approached or reasons for non-participation.

Forty-six women agreed to participate and signed an informed consent form. Responses to the questionnaires were collected through pen-and-paper face-to-face interviews. The research assistant read the questions aloud to the participants and recorded their responses. Each questionnaire was completed one at a time, in the same order for all participants. The questionnaires contained only fixed-response options, with no open-ended questions. The rationale for administering the questionnaires through interviews was to reduce participation barriers among women experiencing homelessness, some of whom may face health-related challenges such as difficulties with concentration or literacy. This approach aimed to facilitate engagement by allowing participants to seek clarification and receive support from the research assistant when needed.

Forty-one women completed the data collection, whereas five discontinued their participation due to concentration difficulties or difficulties understanding the questions, despite assistance from the research assistant. Participants were compensated with a SEK 100 (approximately €10) grocery store voucher after providing informed consent, regardless of whether they completed the data collection.

### Instruments

As part of the larger research project, the women answered questionnaires covering attitudes to homelessness, caring behaviors, experiences of violence, health literacy, psychological distress, spiritual well-being, and use of a mobile phone or the Internet.

This study focused on data collected using the Swedish version of the General Health Questionnaire 12 (GHQ-12) (Goldberg et al., 1997) and the 7-item Questions about Violence (QAV) questionnaire (Björk & Örmon, 2021). We also collected self-reported sociodemographic information. Education level was measured using five categories (not finished primary school, primary school, secondary school, college/university, or other), while length of homelessness was measured using four categories (0‒1 year; over 1 year but less than 5 years; 5 but less than 10 years; and over 10 years).

#### General health questionnaire

The GHQ-12 was developed as a screening tool to detect psychological distress and is used to assess mental well-being by identifying distressing symptoms (Goldberg et al., 1997). The questionnaire provides a general (diagnostically nonspecific) estimation of psychological distress (Lundin & Dalman, 2020). It has been translated into more than 35 languages (Cuellar-Flores et al., 2014) is extensively used, highly reliable, with high sensitivity and specificity, and is convenient for use in clinical settings (Goldberg et al., 1997).

The GHQ-12 comprises 12 statements (alternately phrased as positive or negative) rated on a 4-point scale ranging from 1 (“never”) to 4 (“always”), with total scores ranging from 12 to 48 points. A higher score indicates greater distress, and a total GHQ-12 score >20 points is commonly associated with ongoing anxiety, depression, and/or severe stress reactions (Lundin & Dalman, 2020). The Swedish version of the GHQ-12 has been used in annual national public health surveys since 2004 and has demonstrated high internal consistency (α = .94) and excellent discriminant validity (Lundin et al., 2017).

#### Questions about violence

The QAV questionnaire is adapted from an instrument developed by the WHO for population-based studies aimed at assessing violence against women across different countries (WHO, 2005). Since 2013, the QAV questionnaire has been utilized in clinical practice to identify violence and is recommended for use in Swedish healthcare settings (Björk & Örmon, 2021).

The questionnaire consists of seven yes/no questions addressing experiences of emotional, physical, and sexual violence. It distinguishes between violence perpetrated by someone close and not close to the woman, defining “close” to include partners, family members, and other individuals whom the woman considers important.

Specifically, violence perpetrated by someone close to the woman is assessed with four questions: one on emotional violence, one on physical violence, one on sexual coercion, and one on sexual abuse and violence. Additionally, there is one question covering emotional, physical, and/or sexual violence from someone not close to the woman. Another question assesses whether the woman, during her upbringing, had saw or heard someone close to her being subjected to emotional, physical, and/or sexual violence. Finally, one question explores whether, as an adult, the woman has engaged in emotional, physical, and/or sexual violence toward someone else. The specific wording of each question is provided in Table 2. For the present study, an older version of the QAV questionnaire available at the time of data collection was used. Since then, the QAV has been updated to a newer version (Björk & Örmon, 2021). Hence, the data collected in the present study are based on the older version of the QAV.

In our study, the QAV questionnaire served a dual purpose. First, each of the seven questions was used independently to identify different forms of violence and potential associations with psychological distress. Second, we employed an aggregated score (index) to measure the overall burden of violence in women's lives by summing the “yes” answers, resulting in scores ranging from 0 to 7.

### Statistical Analyses

The reporting of results from the present study follows the *Strengthening the Reporting of Observational Studies in Epidemiology* (STROBE) guidelines for reporting observational studies (von Elm et al., 2014). Categorical data are presented as frequencies and percentages, *n* (%), while ordinal, dichotomous, and continuous data are given as mean values with accompanying standard deviations (SDs). By coding the answers for each of the seven QAV questions as 1 for “yes” and 0 for “no,” an aggregated QAV (index) score ranging from 0 to 7 points was obtained. Associations between experiences of violence measured with the QAV questionnaire and psychological distress measured with the GHQ-12 instrument were estimated using simple (unadjusted) linear regression, with the results presented as slope coefficient β with accompanying 95% confidence intervals (CIs). All statistical analyses were performed using R version 4.2.0 (R Foundation for Statistical Computing, Vienna, Austria), with two-sided *p*-values <.05 considered statistically significant.

### Ethical Approval

This study was approved by the Regional Ethical Board in Stockholm, Sweden (Number 2019-021130) and adhered to the principles of the Declaration of Helsinki (World Medical Association, 2013). All study participants provided written informed consent. They were also informed of their right to withdraw from the study at any time without needing to provide a reason.

Given the potentially distressing nature of the topics covered in the questionnaires, measures were put in place to provide psychosocial support if needed. Participants had access to psychosocial support from a psychologist, social worker, or registered nurse at the PHC in case they experienced distress related to the data collection process. To protect participants’ privacy and autonomy, no medical or social service records were accessed. Additionally, to respect the privacy and autonomy of those who declined participation, no records were kept of these individuals, and reasons for nonparticipation were not collected.

## Results

Characteristics of the study sample are presented in Table 1.

**Table 1.** Characteristics of the Study Sample (*n* = 41).

Variable	Value
Age, *M* (SD)	47.9 (10.4)
Education level, *n* (%)	
Not finished primary school/other	2 (4.9)
Primary school	13 (31.7)
Secondary school	16 (39.0)
College/university	10 (24.4)
Length of homelessness, *n* (%)^a^	
0–1 year^a^	8 (19.5)
>1 year but <5 years	14 (34.1)
5–10 years	13 (31.7)
>10 years	6 (14.6)
Accommodation status	
Roofless	15 (36.6)
Houseless	19 (46.3)
Insecure accommodation	6 (14.6)
Inadequate accommodation	1 (2.4)
GHQ-12 score, *M* (SD)	19.8 (6.8)
0–15 points, *n* (%)	10 (24.4)
16–20 points, *n* (%)	14 (34.1)
21–36 points, *n* (%)	17 (41.5)

*Note*. SD: standard deviation; GHQ-12: General Health Questionnaire 12.

a Including one woman stating that she had experienced homelessness “for periods.”

**Table 2.** Occurrence of Experiences of Violence in the Study Sample Together With Results From Simple (Unadjusted) Linear Regression of Associations Between Experiences of Violence and Psychological Distress.

			Yes	Linear regression	
No.	Form of violence	Question	*n* (%)	β (95% CI)	*P*-value
1	Emotional violence (by someone close)	Have you been threatened, controlled, humiliated, harassed, or anything similar, by someone close to you?	33 (80.5)	7.9 (2.9–12.8)	.**002**
2	Physical violence (by someone close)	Have you been hit, kicked, pushed, or injured in any other way by someone close to you?	32 (78.0)	6.0 (1.1–11.0)	.**017**
3	Sexual coercion (by someone close)	Have you felt pressured to participate in or watch sexual acts against your will by someone close to you?	21 (51.2)	3.5 (−0.7 to 7.7)	.104
4	Sexual abuse and violence (by someone close)	Have you been subjected to verbal, psychological and/or physical sexual abuse by someone close to you?	34 (82.9)	6.5 (1.1–12.0)	.**020**
5	Non-close violence	Have you been subjected to violence according to questions 1–4 by someone who is not close to you?	26 (63.4)	3.2 (−1.2 to 7.7)	.151
6	Witnessing violence against someone close during childhood	During your upbringing, have you seen or heard someone close to you be subjected to violence according to questions 1–4?	24 (58.5)	2.5 (−1.8 to 6.9)	.249
7	Perpetration of violence in adulthood	Have you as an adult subjected someone else to violence according to questions 1–4?	14 (34.1)	3.4 (−1.0 to 7.9)	.132
			*M* (SD)		
		Total QAV (index) score (points)^a^	4.5 (2.1)	1.5 (0.5–2.5)	.**004**

*Note*. CI: confidence interval; SD: standard deviation; QAV: Questions about Violence. Significant *P*-values are given in bold. In total, *n* = 35 (85.4%) answered “Yes” to at least one of Questions 1–4, *n* = 36 (87.8%) answered “Yes” to at least one of Questions 1–4 and 6, *n* = 36 (87.8%) answered “Yes” to at least one of Questions 1–6, and *n* = 13 (31.7%) answered “Yes” to both Question 7 and at least one of Questions 1–6.

a Possible range: 0–7 points.

The 41 women had a mean (SD; range) age of 47.9 (10.4; 26–68) years, with most having either a secondary school (*n* = 16; 39.0%) or college/university (*n* = 10; 24.4%) education. A majority (*n* = 22; 53.7%) had experienced homelessness for <5 years, while 36.6% (*n* = 15) were roofless (i.e., sleeping rough) and 46.3% (*n* = 19) were houseless (i.e., staying in temporary accommodations). The mean (SD; range) GHQ-12 score was 19.8 (6.8; 7–32) points, with 17 (41.5%) women having a score of >20 points, corresponding to a clinical indication of anxiety, depression, and/or severe stress reaction.

### Experiences of Violence

Results regarding the women's experiences of violence are presented in Table 2. A total of 85.4% (*n* = 35) reported having experienced violence perpetrated by someone close to them, such as a family member or intimate partner. Among these women, sexual abuse and violence was the most frequently reported form of violence (82.9%), followed by emotional violence (80.5%), physical violence (78.0%), and sexual coercion (51.2%).

In addition, 63.4% (*n* = 26) of the sample reported experiences of emotional, physical, and/or sexual violence by someone not close to them, such as a stranger or an acquaintance. There was substantial overlap between the two groups: among the 35 women who experienced violence by someone close, 71.4% had also experienced violence by someone not close. Conversely, of the 26 women who had experienced violence by someone not close, 96.2% had also been exposed to violence by someone close to them. These figures indicate that most participants had been exposed to violence from both close and non-close perpetrators, reflecting a high level of cumulative exposure to violence within the sample. Additionally, 58.5% of the women reported having witnessed emotional, physical, and/or sexual violence against someone close to them during childhood. A further 34.1% stated that, as adults, they themselves had perpetrated emotional, physical, and/or sexual violence. Taken together, 87.8% of the women had experienced some form of violence victimization (including witnessing violence), while 31.7% had both experienced and perpetrated violence.

### Associations Between Experiences of Violence and Occurrence of Psychological Distress

Results from linear regressions of associations between experiences of violence (predictor) and the occurrence of psychological distress (outcome) are outlined in Table 2.

Having experienced emotional or physical violence by someone close to the women was significantly associated with a higher level of psychological distress. Women who had experienced emotional or physical violence by someone close to them had on average 7.9 (95% CI: 2.9–12.8; *P* = .002) and 6.0 points (95% CI: 1.1–11.0; *P* = .017) higher GHQ-12 scores, respectively.

Regarding the question about sexual abuse and violence: “Have you been subjected to verbal, psychological and/or physical sexual abuse by someone close to you?,” a significant association was found with the occurrence of psychological distress, with an average of 6.5 (95% CI: 1.1–12.0; *P* = .020) points higher GHQ-12 score for women who answered “yes” to this question. No significant associations between experiences of violence and the occurrence of psychological distress were found for any of the remaining QAV questions.

The mean (SD) aggregated QAV (index) score was 4.5 (2.1) points, that is, on average, each woman answered “yes” to 4.5 out of the seven QAV questions. A higher QAV index score was significantly associated with a higher degree of occurrence of psychological distress, with an average increase of 1.5 (95% CI: 0.5–2.5; *P* = .004) points on the GHQ-12 score for each additional QAV questions answered “yes.”

## Discussion

This study represents a novel attempt to explore different forms of violence and their associations with psychological distress among women experiencing homelessness. The findings suggest that violence perpetrated by someone close, encompassing physical violence, emotional violence, or sexual abuse and violence, is associated with psychological distress among women experiencing homelessness. Furthermore, our study highlights the devastating effects of violence on the women's health and well-being; the more violence in their lives, the worse the psychological distress.

It has been concluded that violence against women has reached epidemic proportions in many societies worldwide (Alhabib et al., 2010; WHO, 2021). In 2018, the lifetime prevalence of intimate partner violence and non-partner sexual violence among women aged 15–49 years in countries and areas of the WHO European Region was 26% (WHO, 2021). Regarding the prevalence in our study, the majority of the women reported exposure to emotional, physical, and sexual violence by someone close to them, with sexual abuse and violence reported by 83%. Additionally, 63% reported exposure to violence by someone not close to them. Consistent with the existing literature (Beijer et al., 2018; Calvo et al., 2022; Riley et al., 2020; Roze et al., 2020), the reality is that violence is embedded in the daily lives of women experiencing homelessness. Notably, most of the women who had experienced violence from someone not close to them had also been subjected to violence by someone close. This finding reinforces the importance of addressing violence exposure in intimate and family relationships, as it appears to be the dominant factor in these women's experiences of violence.

Our findings, which indicate associations between violence perpetrated by someone close to the woman and psychological distress, contribute to the growing body of evidence highlighting the adverse impact of violence in close relationships on women's health (de Souza Cantão et al., 2024; White et al., 2024). However, our study offers an important clinical insight: emotional, physical, and sexual violence perpetrated by someone close to the woman are each independently associated with psychological distress among women experiencing homelessness. Given that all forms of violence pose a significant risk to health and well-being, ensuring a more consistent approach to care—one that avoids minimization and prioritizes listening to women—may serve as a starting point (Beijer et al., 2018; Riley et al., 2020; Tinland et al., 2018). A holistic approach that acknowledges the harmful effects of all forms of violence by someone close is essential for ensuring comprehensive and trauma-informed care.

Our findings did not show a significant association between violence perpetrated by someone not close to the woman and psychological distress among women experiencing homelessness. One possible explanation is that violence from someone close involves a breach of trust, unlike violence from a stranger, where no emotional bond exists (Briere & Jordan, 2004; Platt et al., 2009). Furthermore, the normalization of violence within the context of homelessness may play a role (Brush et al., 2018; Gaber et al., 2024). This is a complex issue shaped by socioeconomic and environmental factors, including exposure to violence during upbringing. It underscores the harsh reality faced by these women, for whom violence is not merely a threat but an anticipated part of everyday life. Yet, the profound impact of non-partner sexual assault on psychological well-being has been consistently highlighted in the literature (Dworkin, 2020; Dworkin et al., 2017), reinforcing the need to further explore its consequences among women experiencing homelessness.

Additionally, it is important to acknowledge the severe social isolation experienced during periods of homelessness, which often results in a reduction in social networks (Omerov et al., 2020; Platt et al., 2009; Roze et al., 2020). Consequently, the significance of maintaining close relationships is magnified, as they serve as critical pillars of support during challenging times. Therefore, it is plausible to suggest that within the context of homelessness, women facing violence from someone close, such as within the microsystem of family or intimate relationships, may endure heightened levels of psychological distress. Future research should specifically explore the impact of violence from both close and non-close relationships on psychological distress among women experiencing homelessness. Exploring this intersectionality is essential for developing targeted interventions that address the unique vulnerabilities of women navigating homelessness. Additionally, future studies should further investigate how different forms of violence (e.g., physical, emotional, and sexual) contribute to psychological distress and specific mental health conditions. Gaining a clearer understanding of how each form of violence affects mental health is crucial for designing interventions that effectively address their distinct consequences and provide tailored support for women experiencing homelessness.

Our study underscores the importance of healthcare professionals recognizing the cumulative impact of violence exposure on women's psychological well-being. We found that increased exposure to violence is associated with more severe psychological distress, and 41.5% of the participating women reported distress indicative of mental ill-health based on GHQ-12 scores. However, the GHQ-12 questionnaire provides a broad assessment of psychological distress without specific diagnostic categorization (Lundin & Dalman, 2020). Therefore, the reported distress levels may encompass overlapping symptoms of anxiety, depression, and posttraumatic stress. While our data do not allow us to determine whether these diagnoses stem from a shared traumatic stress construct, previous research suggests that such overlaps may exist (Dekel et al., 2014). This highlights the potential benefits of integrated treatment approaches. Rather than addressing these conditions in isolation, interventions targeting underlying traumatic stress could offer more effective, simultaneous management of different comorbidities. To refine intervention strategies, future research should incorporate diagnostic-specific measures that differentiate between PTSD, anxiety, and depression, for a deeper understanding of how different forms of psychological distress manifest in contexts of severe adversity. Recognizing these complexities is crucial, as homelessness itself can be a profoundly traumatic experience in which violence and instability exacerbate psychological distress and mental health conditions such as anxiety, depression, and PTSD (Beijer et al., 2018; Duke & Searby, 2019; Li & Urada, 2020; Roze et al., 2020; Tinland et al., 2018; Tweed et al., 2021).

Over half (58.5%) of the women in our study reported witnessing someone close to them being subjected to violence during their upbringing. Additionally, every third (34.1%) woman stated that, as adults, they themselves had perpetrated violence against someone else. These findings underscore the intergenerational impact of violence on women's lives, as evidenced by the fact that nearly one-third (31.7%) of the women were both victims and perpetrators of violence.

Notably, neither witnessing violence during childhood nor perpetrating violence in adulthood was significantly associated with psychological distress in our study. This suggests that while direct experiences of violence are strongly linked to psychological distress, the role of indirect exposure and later perpetration requires further exploration. However, adverse childhood experiences are well-documented risk factors for homelessness, as well as poor health and impaired functioning (Liu et al., 2021). Our findings align with the cycle of violence theory, which posits that exposure to violence in childhood or adolescence can increase the likelihood of engaging in violent behavior later in life, thereby perpetuating the cycle (Widom, 2017). Breaking this cycle is therefore essential for creating safer environments, fostering healthier relationships, and promoting thriving communities.

Addressing violence is a fundamental step in tackling the complex relationship between trauma and mental health issues faced by women experiencing homelessness. To achieve this goal, evidence-informed interventions specifically tailored to support women at risk of, or currently experiencing, homelessness, are crucial. These interventions have been shown to include permanent housing subsidies, post-shelter advocacy, and case management or social support services (Andermann et al., 2021). The implementation of such interventions has demonstrated significant reductions in homelessness, food insecurity, exposure to intimate partner violence, and psychosocial distress among women. Additionally, they have contributed to improvements in self-esteem, quality of life, and child well-being, while also leading to fewer child separations and placements in foster care.

Population-wide measures are essential for preventing women from experiencing homelessness in the first place. These measures include improving access to childcare and stable employment, implementing flexible work conditions, narrowing wage gaps, recognizing and compensating informal family caregiving (often performed by women), challenging social norms that perpetuate intimate partner violence, and ensuring that women have autonomy over household wealth and decision-making (Andermann et al., 2021; WHO, 2021).

Urgent action is needed to implement evidence-informed interventions (Andermann et al., 2021; WHO, 2021) and conduct randomized controlled studies specifically targeting trauma-related conditions among populations experiencing homelessness (Klarare et al., 2024). While some existing interventions that enhance participants’ strengths and skills have shown promise, more robust studies are essential to provide definitive recommendations. Future research should examine the relationship between different forms of violence, psychological distress, and supportive interventions. These steps are crucial for transforming support for women experiencing homelessness, dismantling structural gender inequalities, and ultimately eradicating violence to significantly improve women's health and well-being.

### Strengths and Limitations

The implications of our findings should be interpreted cautiously, considering the convenience sampling method, which was conducted at a single PHC in Stockholm, Sweden. This approach may have limited the diversity of the sample and the broader applicability of the results. Furthermore, data on participants’ country of birth were not collected. Given that the ability to speak and understand Swedish was an inclusion criterion, this information was deemed less relevant. However, in hindsight, this may be considered a limitation.

Additionally, the relatively small sample size (*n* = 41) may have reduced the statistical power to detect certain associations, such as the link between violence from individuals not close to the woman and psychological distress. However, the sample size still exceeded the threshold value determined by the power calculations (Gaber et al., 2022). Furthermore, we lack information on the total number of women approached for participation, which limits our ability to assess potential selection bias. Data collection also partially occurred during the COVID-19 pandemic, a period characterized by increased violence against women, which may have influenced the responses.

Another limitation of the present study relates to the measurement of psychological distress. While the GHQ-12 is a well-established instrument for assessing general psychological distress, it does not distinguish between specific diagnoses such as anxiety, depression, or PTSD (Lundin & Dalman, 2020). Future studies could benefit from incorporating additional diagnosis-specific instruments to provide a more nuanced understanding of mental health outcomes.

Regarding the assessment of violence exposure, the QAV questionnaire is based on the WHO's validated instrument, *Women's Health and Life Experiences*, commonly used in population-based studies to assess exposure to violence (WHO, 2005). A key strength of the QAV is its focus on capturing experiences of violence, as research shows that such targeted questionnaires are more effective in identifying victimization compared to broader ones (Alhabib et al., 2010; WHO, 2005). The QAV also provides detailed descriptions and examples of violent acts, which may help address the normalization or lack of recognition of violent experiences among respondents.

However, we utilized an early clinical version of the QAV questionnaire. Using the later validated and standardized version might have yielded more robust data, as that version has undergone validation through cognitive interviewing and content validity processes (Björk & Örmon, 2021). Furthermore, the QAV questionnaire includes four questions addressing violence perpetrated by someone close to the woman, whereas only one question assesses violence perpetrated by someone not close to her. This imbalance limits our ability to draw conclusions about potential differences between violence perpetrated by individuals with close versus non-close relationships to the woman.

To summarize, despite these limitations, our study highlights the vulnerabilities of an underserved and marginalized group in society, providing valuable insights into the experiences of different forms of violence and their impact on psychological distress among women experiencing homelessness.

## Conclusions

Our study highlights the high prevalence of violence against women experiencing homelessness and its strong association with psychological distress, particularly in the context of close relationships. While direct experiences of emotional, physical, and sexual violence in close relationships were significantly linked to distress, limitations in our measurement tools restrict direct comparisons between violence perpetrated by close and non-close individuals. However, since most women exposed to non-close violence had also experienced violence from someone close, addressing intimate and familial violence remains critical.

Neither witnessing violence in childhood nor engaging in violent behavior in adulthood was significantly associated with distress, suggesting that indirect exposure may play a different role in shaping psychological outcomes.

Structural changes are essential to combat gender-based violence and inequalities. Given the strong link between violence and psychological distress in this population, targeted policies and trauma-informed interventions are urgently needed to improve women's health and well-being.
